# Predictors of fibrogenesis in children with JIA: a single-center pilot study

**DOI:** 10.1186/s12969-023-00937-1

**Published:** 2024-01-02

**Authors:** Olga Pavlova, Natalia Shevchenko, Sergey Pavlov, Tetiana Holovko, Liudmyla Bogmat

**Affiliations:** 1 Department of Pediatrics, School of Medicine, V. N. Karazin Kharkiv National University, Yuvileinyi Avenue, 52-A, Kharkiv, 61153 Ukraine; 2 Cardiorheumatology Department, State Institution “Institute of Health Protection of Children and Adolescents of the National Academy of Medical Sciences of Ukraine”, Kharkiv, Ukraine; 3 Central Scientific Research Laboratory, Kharkiv Medical Academy of Postgraduate Education, Kharkiv, Ukraine

**Keywords:** Juvenile idiopathic arthritis, Methotrexate, Fibrosis, Inflammation, Basic fibroblast growth factor, Vascular endothelial growth factor, Children, Adolescents, Follow-up

## Abstract

**Background:**

Patients with rheumatological diseases are at high risk of developing irreversible fibrotic changes, both articular and extra-articular, as a result of tissue damage caused by the chronic phase of persistent inflammation. Thus, our purpose was to study early markers of fibrosis formation in children with juvenile idiopathic arthritis (JIA).

**Methods:**

Seventy patients with juvenile idiopathic arthritis, namely, polyarthritis (64.29%) and oligoarthritis (35.71%) variant JIA (mean age 13.3 years, 64.29% girls, 35.71% boys), were included in this 4-year prospective study. Basic fibroblast growth factor (bFGF) and vascular endothelial growth factor (VEGF) levels were determined by ELISA kits.

**Results:**

We evaluated bFGF (mean: 7478.21 pg/ml; min: 4171.56 pg/ml; max: 18,011.25 pg/ml) and VEGF (mean: 342.47 pg/ml; min: 23.68 pg/ml; max: 2158.91 pg/ml) levels in children with JIA. Children with JIA had a higher VEGF level when JIA onset occurred after 15 years of age and they had a high disease activity; additionally, a higher bFGF level was observed in children older than 14 years and in those with a JIA onset after 15 years of age, the oligoarticular variant, a moderate disease activity and regardless of MTX administration but more often when MTX was administered at a dosage from 10 to 12.5 mg/m^2^/week.

**Conclusions:**

Laboratory screening of fibrosis formation predictors could help identify patients who may be at greater risk of adverse outcomes. Children with JIA had higher bFGF and VEGF levels when JIA onset occurred after 15 years of age, depending on disease activity.

## Background

Patients with rheumatological diseases are at high risk for poor outcomes when severe fibrosis occurs as a result of tissue damage caused by the chronic phase of persistent inflammation [1]. Organ fibrosis during rheumatoid arthritis (RA) is a result of a dysregulated tissue repair process due to fibroblastic cell types and activation states and inflammatory macrophages [2]. As a result, a common extra-articular manifestation of RA is lung involvement, which is a leading cause of death in patients with RA and is associated with significant morbidity and mortality [3]. Cardiovascular complications are the leading cause of mortality in patients with RA. [4]. Only limited data are available on the risk of fibrosis in patients with RA on long-term methotrexate (MTX) treatment; despite the association of MTX with a range of liver-related adverse events, there are also controversial results in patients with RA [5]. However, in patients with early-onset juvenile idiopathic arthritis (JIA), the duration of the disease and the use of methotrexate can exceed 10 years before these patients reach the age of 18. Thus, there is a need to investigate fibrosis formation predictors in children with JIA to reduce future fibrosis. Fibroblasts are highly dynamic cells that play a central role in tissue repair, wound healing and fibrosis, complex processes involving biochemical and physiological phenomena such as inflammation, proliferation, and remodeling. However, the mechanisms by which they contribute to both physiological and pathological states of extracellular matrix deposition and remodeling are just starting to be understood [6]. Researchers have found that basic fibroblast growth factor (bFGF) and vascular endothelial growth factor (VEGF) are important in different dynamic stages and can improve cell migration, proliferation, differentiation, and angiogenesis [7].

Knowledge about the role of bFGF in pathological and physiological conditions has expanded significantly over the past decades but is still not well understood. bFGF has been shown to be expressed in fibroblasts and adipocytes, to have a close connection with the formation of fibrosis and the inflammatory reaction during angiogenesis, to interact with monocytes/macrophages, and to increase and decrease the concentration of human interleukin-1β. In addition, administration of recombinant bFGF reduces inflammation and fibrotic lung changes in patients with asthma [8]. bFGF also participates in the activation of stellate cells of the liver, fibrogenesis, angiogenesis, and the development of hepatocellular carcinoma [9]. Thus, our purpose was to study early markers of fibrosis formation in children with juvenile idiopathic arthritis.

## Methods

Seventy patients with JIA were included in this 4-year prospective study. The International League of Associations for Rheumatology classification of JIA was used. All children with JIA were divided into groups according to sex, patient age and age at JIA onset, corresponding to the main stages of puberty development. Additionally, all patients were distributed into groups by the variant of arthritis. The active inflammatory process duration, provided that therapy was effective, was considered to be up to one year. Within three years with successful treatment, stable drug-free remission can be achieved. Therefore, all patients were divided into three groups: those with a JIA duration of less than one year, those with a JIA duration from one to three years and those with a JIA duration greater than three years. Disease activity and the Juvenile Arthritis Disease Activity Score (JADAS), namely, JADAS-27, and treatment with methotrexate and its regimen (mg/m^2^ of body surface) were also used for group formation. The exclusion criteria were children who had a systemic variant of the disease or enthesitis-associated arthritis, concomitant endocrine diseases (diabetes, autoimmune thyroiditis, obesity) and gastrointestinal diseases. None of the examined patients had viral hepatitis, tuberculosis or other persistent chronic infections.

Rheumatoid factor (RF), antinuclear antibodies (ANA), erythrocyte sedimentation rate (ESR), C-reactive protein (C-RP), circulating immune complex (CIC) and antistreptolysin-O (ASL-O) were analyzed in this study using standard methods. Levels of basic fibroblast growth factor (bFGF) and vascular endothelial growth factor (VEGF) were evaluated in blood serum with human ELISA kits (Elabscience, USA). According to the study design and the conclusion of the local bioethical committee, biopsies were not collected.

Statistical processing of the obtained research results was carried out using a package of application programs ("Excel for Windows", "Statistica 7.0 for Windows"). Before statistical processing, all data were checked for normality of distribution. The obtained results are presented in the form of mean values (M), standard errors and quartiles. To determine the probability of differences in indicators, parametric (Student's t test, Fisher's angular transformation) and nonparametric criteria (Wilcoxon-Mann‒Whitney U test, dispersion index of Kruskal‒Wallis in case of comparison of more than two points) were used. One-way ANOVA was used to statistically compare the values of the studied groups. Next, the least significant difference test was used. Differences were considered probable at a p value of < 0.05. A study in a group of healthy children was not conducted; the obtained results were assessed focusing on disease manifestation. Correlation and regression were used to assess the relationship between a series of indicators.

Construction of logistic regression models was carried out by the method of step-by-step exclusion of prognostic factors with the determination of the minimum set of predictors based on the estimation of the value of the coefficient of determination R^2^, which shows the share of influence of all predictors of the model on the dependent variable. The critical level of significance for testing statistical hypotheses when comparing groups and for logistic analysis was set at a *p* value of < 0.05.

## Results

Among 70 patients with a mean age of 13.3 years, 64% were female and 36% were male. Fifty-four percent of the patient population was within the age range of 10–13 years old, and 46% were 14–18 years old. Approximately 13% of patients had onset of JIA symptoms during the first three years of life, 17% were symptomatic during 3–5 years of age, 29% during 6–10 years of age, 31% during 11–14 years of age, and 10% during 15–18 years of age. Most of the patient population had polyarthritis (64%), and the remainder had persistent oligoarthritis (36%). Approximately 16% of patients had a JIA duration of less than a year, 37% had a duration of 1–3 years, and the majority of them (47%) had a duration of more than 3 years.

According to the JADAS-27, children in the inactive stage made up 4% of the cohort, and the percentage of children with low activity was 63%, with that of average activity being 27% and high activity being 6%. The majority (74%) of patients were treated with methotrexate; however, 26% of patients were prescribed MTX but had not yet received it. Among patients treated with MTX, 8% had a dosage of less than 10 mg/m^2^/week, 38% had a dosage of 10–12.5 mg/m^2^/week, 37% had a dosage of 12.6–15 mg/m^2^/week, and 17% had a dosage of over 15 mg/m^2^/week. Additionally, folic acid was administered in combination with MTX treatment at a dosage ranging from 0 to 15 mg/week; the average dosage was 5.52 ± 0.49 mg/week.

We evaluated VEGF (min: 23.68 pg/ml; Me: 244.04 pg/ml; max: 2158.91 pg/ml) and bFGF (min: 4171.56 pg/ml; Me: 6809.27 pg/ml; max: 18,011.25 pg/ml) blood serum levels in children with JIA.

The highest mean VEGF level in children with JIA was related to high disease activity according to the JADAS-27 pattern and was significantly higher than that in children with inactive disease activity (*p* = 0.027), low disease activity (*p* = 0.0001), and moderate disease activity (*p* = 0.001). Additionally, a significantly higher mean VEGF level was observed in patients with JIA onset at older than 15 years than in children with JIA onset at 3 to 5 years of age (*p* = 0.010), at 6 to 10 years of age (*p* = 0.022), and at 11 to 14 years of age (*p* = 0.034). Significantly higher mean VEGF levels were observed in patients with JIA durations from 1 to 3 years than in patients with JIA durations of less than 1 year (*p* = 0.036) and JIA durations of greater than 3 years (*p* = 0.049) (Table 1).

**Table 1:** Average levels of VEGF, pg/ml

Indicator		n	means	SD	SE	min	max	Q25	ME	Q75
Sex	Boys	25	296.43	198.12	39.62	24.56	1014.90	215.91	270.77	360.13
	girls	45	368.06	419.59	62.55	23.68	2158.91	128.70	223.65	385.59
Age	10–13 y	38	385.87	453.63	73.59	67.75	2158.91	128.70	216.27	360.13
	14–18 y	32	290.94	179.63	31.76	23.68	824.90	201.85	260.93	366.30
Age of JIA onset	< 3 y	9	341.57	300.01	100.00	55.84	1042.60	177.23	218.73	440.39
	3–5 y	12	**227.11**	213.74	61.70	23.68	824.90	111.80	170.20	277.80
	6–10 y	20	**304.64**	229.28	51.27	24.56	1014.90	129.11	286.25	370.16
	11–14 y	22	**337.83**	430.62	91.81	45.90	2158.91	184.97	234.55	293.98
	15–18 y	7	**664.11**	538.89	203.68	101.52	1422.60	316.48	534.53	1422.60
Variant	oligo	25	331.97	407.48	81.50	24.56	2158.91	128.70	293.98	360.13
	poly	45	348.31	329.66	49.14	23.68	1422.60	169.49	244.04	380.19
Duration	< 1 y	11	**203.92**	135.37	40.82	73.71	495.95	82.32	215.91	293.98
	1–3 y	26	**469.97**	482.99	94.72	45.90	2158.91	223.65	309.10	534.53
	˃ 3 y	33	**288.21**	251.53	43.785	23.680	1042.60	128.70	219.43	342.38
Disease activity according to JADAS-27	inactive disease	3	**418.60**	173.44	100.13	301.72	617.88	301.72	336.21	617.88
	low	44	**276.22**	338.33	51.01	23.68	2158.91	115.11	193.06	306.99
	moderate	19	**349.67**	235.99	54.14	24.56	1014.90	223.65	272.18	534.53
	high	4	**979.94**	562.50	281.25	249.67	1422.60	537.29	1123.75	1422.60
MTX treatment	not present	18	400.71	393.11	92.66	82.32	1422.60	223.65	293.98	360.13
	present	52	322.31	344.75	47.81	23.68	2158.91	147.34	222.25	366.30
Dosage of MTX, mg/m^2^/week	< 10	4	319.32	166.06	83.03	101.52	495.95	200.57	339.90	438.07
	10–12,5	20	402.46	464.19	103.79	23.68	2158.91	208.88	260.93	369.00
	12,6–15	19	301.33	284.31	65.23	24.56	1042.60	92.25	215.91	440.39
	˃ 15	9	189.83	129.90	43.30	55.84	509.85	128.70	169.49	184.97
All children with JIA		70	342.47	356.53	42.61	23.68	2158.91	165.98	244.04	360.13

**Significant difference was obtained only in the following groups:**

Age of JIA onset

(3–5 y. 15–18 y. *p* = 0.010; 6–10 y. 15–18 y. *p* = 0.022; 11–14 y. 15–18 y. *p* = 0.034)

Duration

(< 1 y. 1–3 y. *p* = 0.036; 1–3 y. ˃ 3 y. *p* = 0.049)

Disease activity according to JADAS-27

(inactive disease high *p* = 0.027; low high *p* = 0.001; moderate high *p* = 0.001)

The average level of bFGF significantly increased in children with an age of JIA onset from 15 to 18 years versus an age of JIA onset up to 3 years (*p* = 0.003), 3–5 years (*p* = 0.025), and 11–14 years (*p* = 0.001) and in children with an age of JIA onset from 6 to 10 years versus an age of JIA onset from 11–14 years (*p* = 0.041). Higher bFGF levels were also found in patients with moderate disease activity according to the JADAS-27 pattern versus those with low disease activity (*p* = 0.0004) (Table 2).

**Table 2:** Average levels of bFGF, pg/ml

Indicator		n	means	SD	SE	min	max	Q25	ME	Q75
Sex	Boys	25	7551.88	3062.34	612.47	4808.33	18,011.25	4988.39	7300.22	8331.84
	girls	45	7437.28	2643.30	394.04	4171.56	17,119.83	5649.78	6787.45	8556.67
Age	10–13 y	38	6968.45	1914.40	310.56	4171.56	14,361.1	5559.70	6639.79	7632.24
	14–18 y	32	8083.54	3480.40	615.25	4225.58	18,011.25	5530.27	7730.35	8662.84
Age of JIA onset	< 3 y	9	**6217.65**	773.18	257.73	4924.41	7471.25	5763.66	6109.17	6787.45
	3–5 y	12	**7475.90**	1686.40	486.82	4171.56	10,505.88	6528.54	7276.15	8721.77
	6–10 y	20	**8148.82**	3213.02	718.45	4808.33	18,011.25	6335.87	7393.79	8840.89
	11–14 y	22	**6488.29**	1606.85	342.58	4225.58	9205.14	4988.39	5594.91	8016.54
	15–18 y	7	**10,297.98**	4928.90	1862.95	5559.70	17,119.83	5559.70	8331.84	17,119.83
Variant	oligo	25	7552.63	2765.49	553.09	4808.33	18,011.25	5530.27	7453.78	8697.72
	poly	45	7436.86	2816.12	419.80	4171.56	17,119.83	5559.70	6787.45	8310.03
Duration	< 1 y	11	7300.57	2818.21	849.72	4988.39	14,361.11	4988.39	6563.66	9125.10
	1–3 y	26	7812.12	3196.07	626.80	4808.33	17,119.83	5559.70	7483.02	8579.85
	˃ 3 y	33	7274.33	2457.76	427.84	4171.56	18,011.25	5959.60	6787.45	8310.03
Disease activity according to JADAS-27	inactive disease	3	6743.29	1112.43	642.26	5959.60	8016.54	5959.60	6253.74	8016.54
	low	44	**6727.11**	1979.52	298.42	4171.56	14,361.1	5420.24	6379.58	7551.74
	moderate	19	**9364.85**	3764.04	863.53	5530.27	18,011.25	7524.14	8331.84	8745.82
	high	4	7329.86	2045.81	1022.90	5559.70	9205.14	5559.70	7277.30	9100.02
MTX treatment	not present	18	7386.12	3806.80	897.27	4808.33	17,119.83	4988.39	5559.70	8331.84
	present	52	7510.08	2368.693	328.48	4171.56	18,011.25	5982.24	7317.01	8444.26
Dosage of MTX, mg/m^2^/week	< 10	4	7610.33	1889.73	944.87	5453.17	10,063.10	6453.48	7462.52	8767.18
	10–12,5	20	7875.05	2291.38	512.37	4988.39	14,361.11	5744.94	7764.70	8651.25
	12,6–15	19	7677.30	2869.74	658.36	4225.59	18,011.25	6253.74	7300.22	8745.83
	˃ 15	9	6301.49	1175.63	391.88	4171.56	8697.72	6004.87	6180.64	6536.69
All children with JIA		70	7478.205	2778.57	332.10	4171.56	18,011.25	5559.70	6809.27	8331.84

**Significant difference was obtained only in the following groups:**

Age of JIA onset

(< 3 y. 15–18 y. *p* = 0.003; 3–5 y. 15–18 y. *p* = 0.025; 6–10 y. 11–14 y. *p* = 0.041; 11–14 y. 15–18 y. *p* = 0.001)

Disease activity according to JADAS-27

(low moderate *p* = 0.0004)

Levels of circulating immune complex (CIC) and antistreptolysin-0 (ASL-O) were associated with VEGF levels. Mostly VEGF correlated with CIC (among: girls *r* = 0.53, patients with oligoarthritis *r* = 0.55, inactive disease activity according to JADAS-27 *r* = 0.47, dosage of MTX, 10–12.5 mg/m^2^/week *r* = 0.67 *p* < 0.05) and with ASL-O (among: girls *r* = 0.47, inactive disease activity according to JADAS-27 *r* = 0.51, MTX treatment *r* = 0.36, dosage of MTX, 10–12,5 mg/m^2^/week *r* = 0.90 *p* < 0.05).

Activity according to the JADAS-27 pattern and the levels of ESR, CIC and ASL-O were associated with bFGF levels. Mostly bFGF was correlated with ASL-O (among boys *r* = 0.45, patients with oligoarthritis *r* = 0.49, moderate disease activity according to JADAS-27 *r* = 0.58, MTX treatment *r* = 0.38, dosage of MTX, 12.5–15 mg/m^2^/week *r* = 0.50 *p* < 0.05), CIC (among patients with polyarthritis *r* = 0.37, low disease activity according to JADAS-27 *r* = 0.34, dosage of MTX, less than 10 mg/m^2^/week *r* = 0.96 *p* < 0.05), and ESR (among girls *r* = 0.37, patients with polyarthritis *r* = 0.38, *p* < 0.05).

In the general group of patients, logistic regression was performed to determine the factors that affect bFGF and VEGF levels and have a negative prognostic value.

$$\mathrm{VEGF}=245.1758+111.614\;(\mathrm{JADAS}\;27)\;+\;108.0411\;\left(\mathrm{ANA}\right)-\;16.3435(\mathrm{RF})\;-\;0.0233\;(\mathrm{MTX}\;\mathrm{dose})$$

*p* = 0.040677

$$\mathrm{bFGF}=4611.25\;+\;846.64\;(\mathrm{JADAS}\;27)\;+\;131.35(\mathrm{ESR})\;-\;1058.32\;\left(\mathrm{ANA}\right)+\;4.3\;\left(\mathrm{ASL}-0\right)$$

*p* = 0.000333

## Discussion

It is clear that the persistence of inflammation in rheumatoid joints is a consequence of an imbalance between angiogenic mediators such as cytokines and growth factors. While various studies, including in vitro studies and tumor models, have focused on the relationship between VEGF and bFGF [10–12], no study has directly focused on the role of growth factors in JIA patients.

Meta-regression analysis of VEGF in RA patients adjusted for age and female sex demonstrated that neither age (*p* = 0.409) nor sex (*p* = 0.757) had a significant effect [10]. In our study of VEGF levels in patients with JIA, we also did not find any significant differences in age (*p* = 0.270) or sex (*p* = 0.425). According to a meta-analysis of RA, there was a positive, weak correlation between circulating VEGF levels and disease activity (DAS-28; ES 0.33, 95% CI 0.22–0.44, *P* < 0.0001, pooled *r* = 0.32), ESR (ES 0.35, 95% CI 0.18–0.51, *P* < 0.0001; summary *r* = 0.34), and CRP (CRP; ES 0.38, 95% CI 0.24–0.52, *P* < 0.0001; summary *r* = 0.36) [13]. According to our data, VEGF levels correlated with CIC and ASL-O, but we also observed that the highest mean VEGF level in children with JIA was related to high disease activity according to the JADAS-27 pattern.

bFGF is the most studied factor in oncological diseases. The results of a meta-analysis revealed that acute myeloid leukemia patients probably have higher circulating levels of bFGF (SMD = 1.15, 95% CI: 0.35–1.94) [14] and that bFGF overexpression has an adverse impact on the survival of patients with lung cancer (HR = 1.202, 95% CI, 1.022–1.382) [15]. The significance of high bFGF levels in inflammatory arthropathies has been insufficiently investigated.

According to our data, the highest mean bFGF level in children with JIA was related to a JIA onset of later than 15 years old and moderate disease activity according to the JADAS-27 pattern. Additionally, in patients with JIA onset at older than 15 years, a significantly higher mean VEGF level was observed. Our data also indicated that both bFGF and VEGF correlated with CIC and ASL-O and that only bFGF had significant correlations with the ESR.

Our studies confirmed the interrelation of VEGF and bFGF levels with indicators of disease activity. According to the logistic regression results, the most significant among them were JADAS 27, ANA and RF. Additionally, an insufficient dose of methotrexate may be a factor contributing to an increase in VEGF.

Thus, the levels of VEGF and bFGF can be considered informative indicators of a poor outcome due to a chronic inflammatory process.

## Conclusions

Vascular endothelial growth factor was more often increased in those with a JIA onset of older than 15 years and with high disease activity. Fibroblast growth factor was more often high in boys, in children older than 14 years and in those with a JIA onset after 15 years of age, those with the oligoarticular variant, those with a medium disease activity and regardless of MTX administration but more often when MTX was administered at a dosage from 10 to 12.5 mg/m^2^/week. The bFGF level was highest in children older than 15 years against a background of an average degree of disease activity. Laboratory screening could help identify patients who may be at greater risk of adverse outcomes.

## Data Availability

The raw data supporting the conclusions of this article will be made available by the authors, without undue reservation.

## Abbreviations

RA: Rheumatoid arthritis
JIA: Juvenile idiopathic arthritis
MTX: Methotrexate
JADAS: Juvenile Arthritis Disease Activity Score
bFGF: Basic fibroblast growth factor
VEGF: Vascular endothelial growth factor
RF: Rheumatoid factor
ANA: Antinuclear antibodies
ESR: Erythrocyte sedimentation rate
C-RP: C-reactive protein
CIC: Circulating immune complex
ASL-O: Antistreptolysin-O
